# Anatomical Background of the Perforator Flap Based on the Deep Branch of the Superficial Circumflex Iliac Artery (SCIP Flap): A Cadaveric Study

**Published:** 2010-01-18

**Authors:** Raphael Sinna, Hassene Hajji, Quentin Qassemyar, David Perignon, Thomas Benhaim, Eric Havet

**Affiliations:** ^a^Department of Plastic, Reconstructive and Aesthetic Surgery, Amiens North Hospital, University of Picardie, place Victor Pauchet, 80054 Amiens, France; ^b^Department of Anatomy, University of Tunis, Tunis, Tunisia; ^c^Department of Anatomy, University of Picardie, Amiens, France

## Abstract

**Background:** The groin flap, based on the superficial circumflex iliac artery, was the first successful free flap. However, its popularity was lost essentially due to variable arterial anatomy. Clinical applications of perforator flap based on superficial circumflex iliac artery suggest that a dominant perforator based on his deep branch is enough to supply a large groin flap. **Methods:** Fresh cadaveric dissections were performed and the perforators of Sartorius based on the deep branch of superficial circumflex iliac artery were identified. The dominant perforator was isolated and injected selectively with methylene blue solution. **Results:** We performed 20 dissections of superficial circumflex iliac artery and the deep branch. We found a deep branch with 2 perforators penetrating the Sartorius muscle. The diameter of the dominant perforator of the deep branch was 0.85 mm on average and the mean injected skin area was 162 cm^2^. **Conclusions:** These findings are in agreement with early clinical suggestion. The dominant musculocutaneous perforator of the deep branch of superficial circumflex iliac artery provides constant and reliable blood supply to ensure a relatively large perforator groin flap.

The groin flap (based on the superficial circumflex iliac artery [SCIA]) was the first-ever successful free flap^1^ described by McGregor and Jackson^2^ in 1972. Concealment of the donor site scar and a large cutaneous flap meant that this procedure quickly became popular. However, the subsequent development of new flap techniques highlighted major shortcomings of the groin flap, such as arterial anatomical variation, a short pedicle, and the bulkiness of the flap itself.

More recently, the development of perforator flaps (PFs) has enabled the use of thinner flaps,^3^,^4^ whereas progress in imaging provides more reliable identification of vessels capable of supplying flaps.^5^^-^7 Thus, the PF based on the SCIA has been described for local or distant coverage of wounds.^8^,^9^ The results of these clinical applications suggest that just 1 dominant perforator from the deep branch of the SCIA may be enough to supply a large groin flap.^9^ However, the anatomical basis for SCIA groin flaps is less well established than for more popular PFs. The aim of the present study was to determine the size, location, and reliability of perforators arising from the deep branch of the SCIA (Fig 1).

## MATERIAL AND METHODS

**Figure 2:**
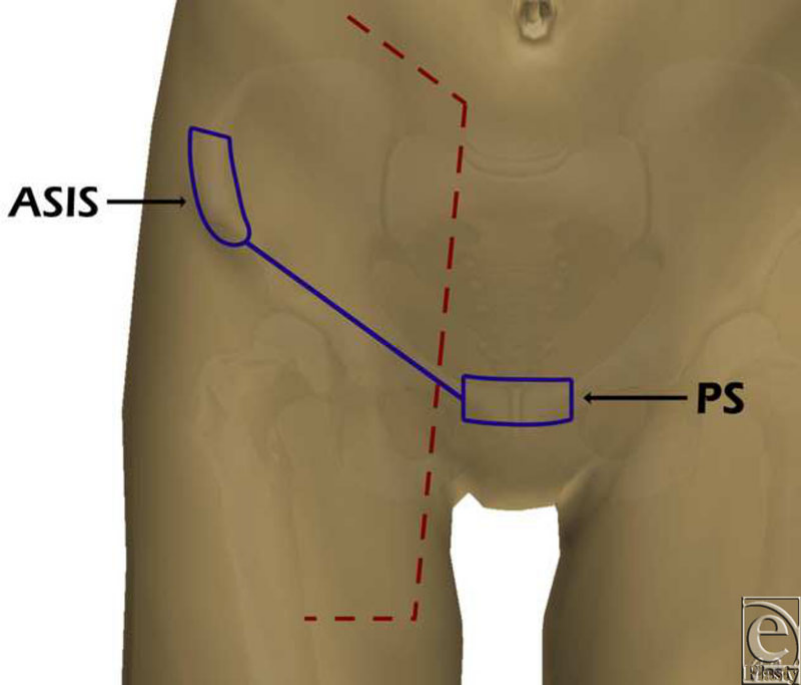
Skin benchmarks and dissection markings. ASIS indicates anterosuperior iliac spine; PS, pubic symphysis.

Ten fresh cadavers (7 men and 3 women) were dissected bilaterally. Cadavers with scars in the groin region or without the deep branch of the SCIA were excluded from the study. The anterosuperior iliac spine, the pubic symphysis, and the crural arcade were used as skin benchmarks (Fig 2).

After the identification of the origin of the SCIA, dissection was initiated medially and then continued laterally to identify the superficial and deep branches of the artery. The deep branch was individualized and its musculocutaneous perforators were identified.

**Figure 3:**
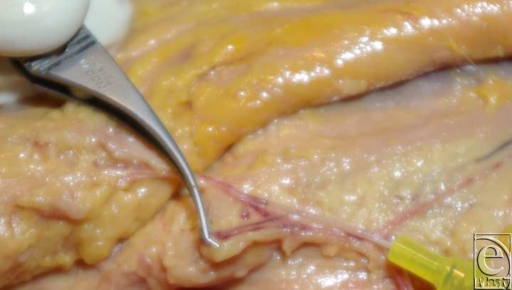
Selective injection of the major perforator of the deep branch with methylene blue solution with a 0.65-mm diameter catheter.

The superficial branch and other perforators were identified and ligated. After identification, the major musculocutaneous perforator of the deep branch was injected with 3 mL of 0.1% methylene blue solution (Fig 3). The location and surface area of the stained skin island were recorded.

**Figure 4:**
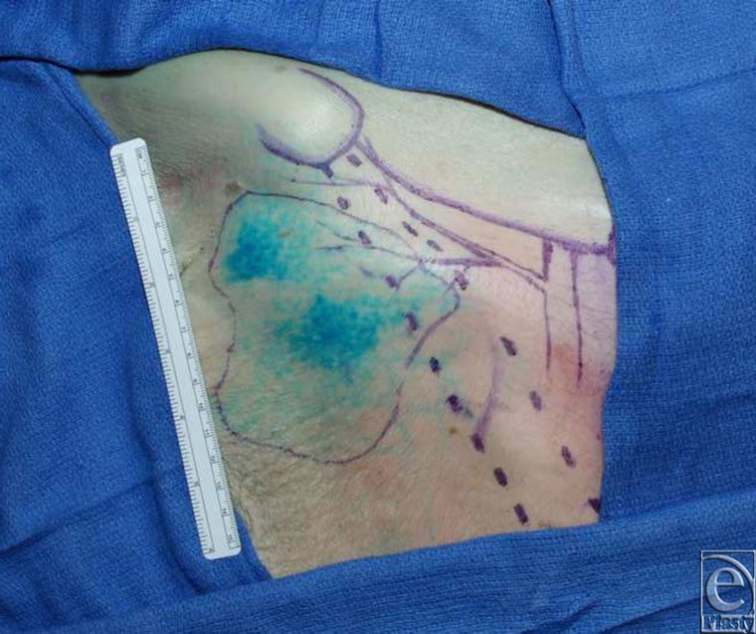
Skin area after selective injection of the dominant perforator.

The following parameters were measured: diameter of the SCIA, diameter of the deep branch of the SCIA, number of musculocutaneous perforators originating from the deep branch, diameter of the major musculocutaneous perforator and the vena comitans, length of the pedicle (ie, the distance between the origin of the deep branch and the major musculocutaneous perforator), and injected skin area (Fig 4).

## RESULTS

**Figure 5:**
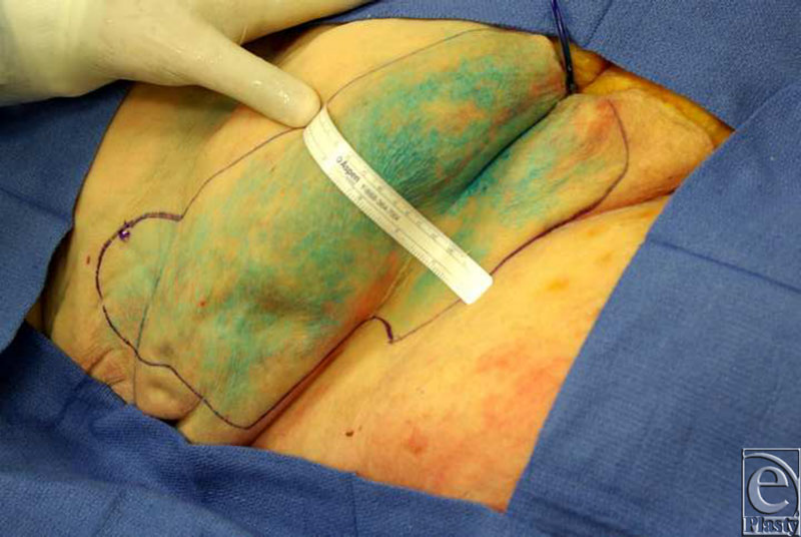
The maximum skin area (375 cm^2^ staining obtained after selective injection.

The 10 bilaterally dissected fresh cadavers yielded 20 SCIA dissections (1). The mean ± standard deviation diameter of the SCIA was 1.92 ± 0.6 mm, with a mean diameter of the deep branch of the SCIA 1.35 ± 0.41 mm. We always found at least 2 musculocutaneous perforators through the sartorius muscle (mean number = 2.37 ± 0.51). The mean diameter of the major musculocutaneous perforator was 0.85 ± 0.12 mm. On average, the vena comitans measured 0.73 ± 0.21 mm in diameter. The mean pedicle length was 4.8 ± 1.3 cm, and the mean surface area of the skin island was 162 ± 50 cm^2^ (Fig 5).

## DISCUSSION

**Figure 6:**
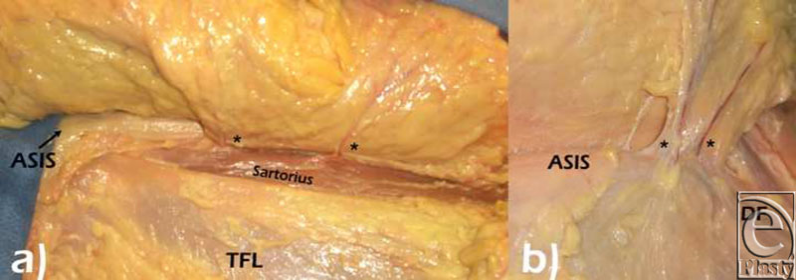
(*a*) The deep fascia was raised to demonstrate the perforator through Sartorius muscle. (*b*) View of the perforators above the deep fascia. ASIS indicates anterosuperior iliac spine; TFL, tensor fasciae; DF, deep fascia; and asterisk (*), perforator vessel.

**Figure 7:**
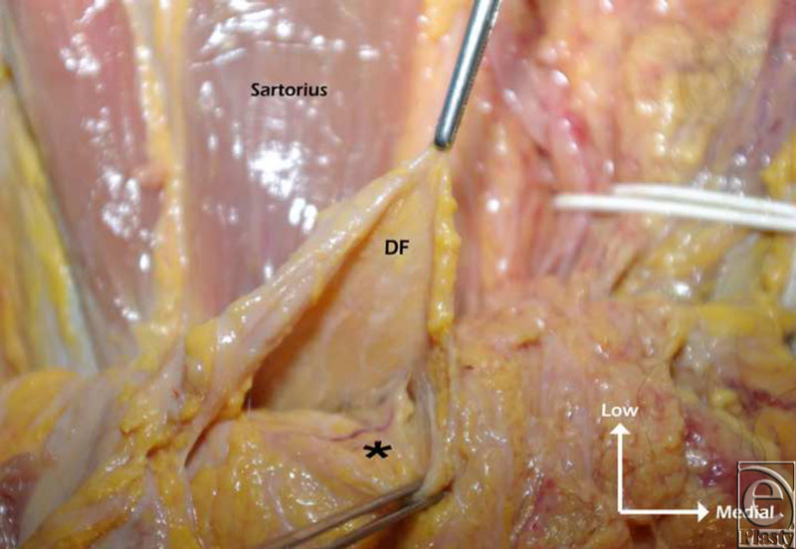
Top view. A musculocutaneous perforator penetrating the deep fascia and running through the subcutaneous tissue. The deep fascia was raised from the Sartorius muscle. Asterisk (*) indicates dominant perforator; DF, deep fascia.

As the first-ever successful free flap^1^ to be described (in 1972),^2^ the groin flap is of historical importance. However, its use was progressively supplemented by more reliable techniques. One of the main reasons for this drop in popularity is the anatomical variability of the pedicle of the groin flap. Although there are many anatomical variations on the trunk of the SCIA and its superficial branch,^10^^-^12 Salmon^13^ described the existence of a perforator artery arising from the SCIA on the medial border of the sartorius muscle. More recently, the development of PF techniques has reduced the donor site morbidity observed with “conventional” flaps. Hence, Koshima et al^9^ reported a large groin flap based on a single, dominant perforator arising from the deep branch of the SCIA. The aim of the present study was to prove the ubiquitous existence of a dominant, musculocutaneous perforator of the deep branch of the SCIA that can be used safely to harvest a reliable PF (Figs 6*a*, *b*, and 7) when the deep branch is found. Although many anatomical studies have described perforators in this region,^14^,^15^ none has described those of the deep branch of the SCIA in detail.

In 20 dissections of the SCIA, we always found a dominant perforator through the Sartorius muscle. The mean diameter (0.85 mm) was similar to that observed for the dominant perforator of a thoracodorsal PF.^16^ The vena comitans was always smaller (with a mean diameter of 0.73 mm). These findings are in agreement with clinical applications described by Koshima et al^9^ and Hsu et al.^8^ These authors reported that a single, dominant perforator of the deep branch is enough to nourish a relatively large groin flap.^9^ We found that the skin surface area was 162 cm^2^ on average, with a maximum recorded value of 375 cm^2^. The selective injection of the dominant perforator of the deep branch of SCIA is in agreement with these clinical conclusions. Therefore, for clinical application, the dissection of the flap should be started from lateral to medial until the Sartorius perforator, above the fascia. But imaging (computed tomographic scan or color Doppler) of the region is necessary preoperatively to confirm the presence of the deep branch. Although the pedicle is short, microdissection would, nevertheless, yield a pedicle with an average length of 7 cm.^17^ When combined with a “worm-eaten defatting” method, this microdissected flap technique enables a uniformly thin groin flap to be transferred without excess accompanying fatty tissue^18^ and to avoid harvesting the flap on the classical superficial branch as described by McGregor and Jackson.^2^

## CONCLUSION

The present anatomical study confirmed our clinical suspicions: a single, dominant perforator arising from the deep branch of the SCIA is capable of supplying a large groin flap. Our results suggest that this type of flap could be useful for the reconstruction of the legs with a thin flap and a well-hidden donor site. The SCIA deep branch perforators can provide a constant, reliable blood supply to a relatively large groin flap.

## Figures and Tables

**Figure 1:**
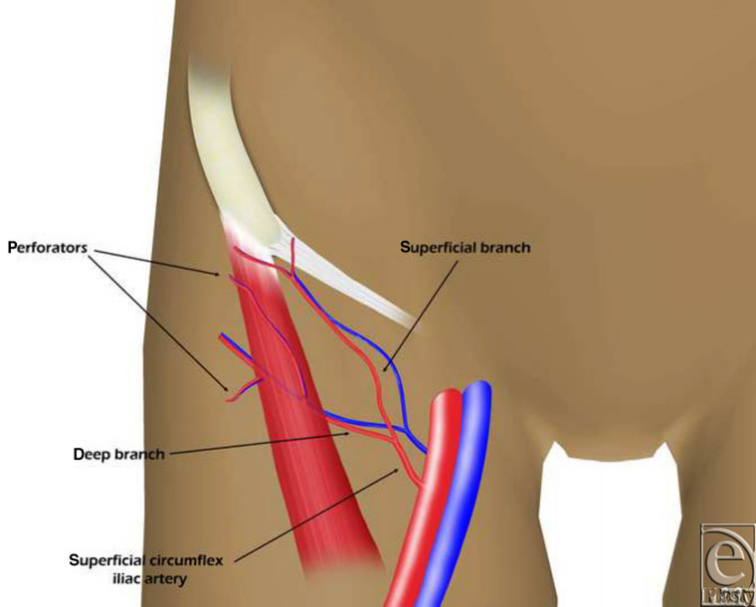
The anatomical basis.

**Table 1:** Results of dissections

	Mean±SD	Range
Superficial circumflex iliac artery diameter, mm	1.92±0.6	1.2–3
Deep branch diameter, mm	1.35±0.41	1–2
Number of Sartorius perforator, *n*	2.37±0.51	2–3
Major perforator diameter, mm	0.85±0.12	0.7–1
Concomitant vein diameter, mm	0.73±0.21	0.6–1
Pedicle length, cm	4.8±1.3	3–8
Injected skin area, cm^2^	162±50	75–375
